# Cu_2_ZnSnSe_4_ nanocrystals capped with S^2−^ by ligand exchange: utilizing energy level alignment for efficiently reducing carrier rec ombination

**DOI:** 10.1186/1556-276X-9-262

**Published:** 2014-05-24

**Authors:** Xia Wang, Dong-Xing Kou, Wen-Hui Zhou, Zheng-Ji Zhou, Si-Xin Wu, Xuan Cao

**Affiliations:** 1 The Key Laboratory for Special Functional Materials of MOE, Henan University, Kaifeng 475004, China; 2 Institute of Oceanographic Instrumentation, Shandong Academy of Sciences, Qingdao 266061, China

**Keywords:** One-step synthesis, Ligand exchange, CZTSe NCs, Energy level alignment

## Abstract

In this work, we employed a convenient one-step synthesis method for synthesizing Cu_2_ZnSnSe_4_ (CZTSe) nanocrystals (NCs) in an excess selenium environment. This excess selenium situation enhanced the reaction of metal acetylacetonates with selenium, resulting in the burst nucleation of NCs at relatively low temperatures. The phase morphology and surface and optoelectronic properties of NCs before and after ligand exchange were discussed in depth. It was found that pure tetragonal-phase structure CZTSe NCs with approximately 1.7-eV bandgap could be synthesized. The removal of large organic molecules on CZTSe NCs after ligand exchange by S^2−^ decreased the resistivity. The bandgap of the films after ligand exchange by 550°C selenization was also decreased due to better crystallinity. For potential application in CZTSe solar cells, we constructed an energy level diagram to explain the mutual effect between the absorption layer and CdS layer. Using cyclic voltammetry (CV) measurement, we found that the highest occupied molecular orbital (HOMO) and lowest unoccupied molecular orbital (LUMO) energy levels of CZTSe films shifted down after ligand exchange. After energy level alignment at the CdS/CZTSe interface, a type I band alignment structure was more conveniently formed after ligand exchange. This structure acted as the barrier against injection electrons from ZnO to the CZTSe layer, and recombination would subsequently be depressed.

## Background

Currently, nontoxic and earth-abundant I_2_-II-IV-VI_4_ quaternary compounds such as Cu_2_ZnSnS_4_ and Cu_2_ZnSnSe_4_ (CZTSe) have been considered as the most promising ‘next-generation’ photovoltaic materials to substitute for CIGSe absorber materials, due to their excellent properties such as high absorption coefficients (1 × 10^5^ cm^−1^) [1-3], suitable absorption bandgap for the solar spectrum, high radiation stability, and considerable cell efficiency [4-6]. Various methods have been used for the preparation of CZTSe materials, including physical methods [7-10] and wet chemical routes [11-15]. Wet chemical routes are more prevalent due to their convenient operability, achievable by using traditional instruments, and low cost. CZTSe nanocrystals (NCs) are usually covered with long alkyl chain ligands to shield the surface of the NC, which can realize homogeneous nucleation and enable easy solution processibility for fabrication. However, these ligands also act as an insulating layer that impedes efficient charge transfer and the dissociation of photogenerated excitons and lead to poor conductivity, limiting NC application in electronic and photoelectronic devices [16-18]. Many results had been recently published regarding the development of new ligand strategies to minimize interparticle spacing. Zhang et al. reported that optical absorption of NCs could be effectively improved after ligand removal [19]. Lauth et al. reported that 3 orders of magnitude conductivity increase of CIGS NC films could be achieved after ligand removal and conductivity enhancement depends on the NC size accentuating the role of trap states and internal grain boundaries in ligand-free NC solids for electrical transport [20]. Carrete et al. and Stolle et al. performed ligand exchange on CZTSe nanoparticles, finding that crystallization of NCs and cell performances could be promoted [21,22]. Their works focused on improving the optical and electrical properties of CZTSe films to increase the photocurrent of the device, but there is no detailed study clarifying the band alignment between the CdS layer and the absorption layer after ligand exchange.

Herein, we employed a convenient one-step method to synthesize CZTSe NCs. The key feature of this synthesis was to use excess Se relative to Cu, Zn, and Sn and conduct the reaction at a relatively low temperature. All-inorganic CZTSe NCs were obtained by ligand exchange strategy using a simple metal-free chalcogenide compound [(NH_4_)_2_S] as the inorganic ligand. We showed the energy level movement of CZTSe films before and after ligand exchange. Using cyclic voltammetry (CV) measurements, we found that the highest occupied molecular orbital (HOMO) and lowest unoccupied molecular orbital (LUMO) energy levels of CZTSe films shifted down after ligand exchange. Utilizing energy level alignment at the CdS/CZTSe interface, we constructed an energy level diagram to explain the physical mechanism of reducing recombination in CZTSe solar cells. This provides a different approach to the design of the absorption layer, which is generally not afforded by previous reports applying interface passivation and the control of trap states, focuses on the problem of recombination, and holds for a more convenient way to optimize interface properties.

## Methods

Cupric(II) acetylacetonate [Cu(acac)_2_], zinc(II) acetylacetonate [Zn(acac)_2_], tin(IV) chloride tetrahydrate (SnCl_4_ · 4H_2_O), 2,4-pentanedione, triethylamine, perchlorethylene 1-dodecanethiol (DDT), and oleylamine (OLA) were purchased from Alfa Aesar (Ward Hill, MA, USA). Tetrabutylammonium hexafluorophosphate (TBAPF_6_) and sodium hydroxide (NaOH) were purchased from Aldrich (St. Louis, MO, USA). Toluene, *N*,*N*-dimethylformamide (DMF), and ethanol are of analytical grade. All water used was obtained from a Millipore Milli-Q purification system (Darmstadt, Germany). The chemicals were used in an as-received condition without further purification.

For the synthesis of Sn(acac)_4_, 20 mmol SnCl_4_ · 4H_2_O was dissolved in 15 ml deionized water. Under magnetic stirring, 90 mmol of 2,4-pentanedione (9 ml) was added and kept stirred for another 15 min. Then Sn(acac)_4_ was precipitated by the addition of triethylamine (6 ml). The resulting Sn(acac)_4_ was washed for several times by ethanol and water, then dried in the vacuum.

A typical synthetic procedure of CTZSe NCs is briefly described as follows: 1 ml OLA, 1 ml DT, and 2 mmol Se powder were placed in a three-neck flask and stirred to dissolve the Se powder. Once the Se powder was completely dissolved, 0.5 mmol Cu(acac)_2_, 0.25 mmol Zn(acac)_2_, 0.25 mmol Sn(acac)_4_, 1 ml DT, and 10 ml OLA were added under vigorous stirring. Then the mixture was placed in an oil bath at 240°C and maintained for 0.5 h. After that, the flask was rapidly cooled to room temperature, and the as-synthesized NCs were separated by precipitation with ethanol and collected by centrifugation at 9,500 rpm for 4 min. The supernatant was decanted. The precipitates were dispersed in hexane and further purified by ethanol for several times. The precipitates were dried under vacuum at room temperature.

The ligand exchange process was carried out according to the literature with some modification [23]. Colloidal dispersion of CZTSe NCs with organic ligand was prepared in toluene, while the solution of CZTSe NCs with inorganic ligand was prepared in polar formamide (FA) immiscible with toluene. For a typical ligand exchange, 20 mg CZTSe NCs was dispersed into 3 ml toluene and 0.1 ml (NH_4_)_2_S was dissolved into 3 ml FA. Then the (NH_4_)_2_S solution in FA was mixed with the CZTSe NC dispersion in toluene. The mixture was stirred for about 10 min leading to a complete phase transfer of CZTSe NCs from toluene to the FA phase. The phase transfer can be easily monitored by the color change of toluene (black to colorless) and FA (yellow to black) phases. The FA phase was separated out followed by triple washing with toluene to remove any remaining nonpolar organic species.

The morphology of CZTSe NCs was characterized by transmission electron microscopy (TEM; JSM-2010, JEOL Ltd., Akishima-shi, Japan). The phase and crystallographic structure of the products were identified by X-ray diffraction (XRD; X'Pert Pro, Philips, Amsterdam, The Netherlands). The UV-visible (UV-vis) absorption spectra were obtained by using a UV-vis spectrometer (Lambda 35, PerkinElmer, Waltham, MA, USA). Fourier transform infrared (FTIR) spectra were recorded on a Nicolet 360 FTIR spectrometer (Thermo Fisher Scientific, Inc., Waltham, MA, USA) using KBr pellets in the range of 4,000 of 400 cm^−1^. The Raman spectrum was recorded using a LABRAM-1B confocal laser micro-Raman spectrometer (HORIBA, Kyoto, Japan) with the wavelength of 632.8 nm. The resistivity was tested by the four-probe method on a digital source meter (Keithley 2400, Keithley Instruments, Inc., Cleveland, OH, USA). Mott-Schottky plots and CV curves were measured by using an electrochemical test station (Novocontrol Technologies, Montabaur, Germany).

## Results and discussion

The successful synthesis of high-quality monodisperse quantum dots (QDs) must start with a swift and short nucleation from supersaturated reactants, followed by growth without further nucleation [24,25]. In this study, this excess selenium situation significantly enhanced the reaction of the metal acetylacetonates [Cu(acac)_2_, Zn(acac)_2_, and Sn(acac)_4_] with selenium, resulting in a short nucleation stage. This synthetic tactic is advantageous over the typical hot-injection synthesis [24], which requires a relatively high injection temperature (usually above 250°C) to generate burst nucleation.

**Figure 1:**
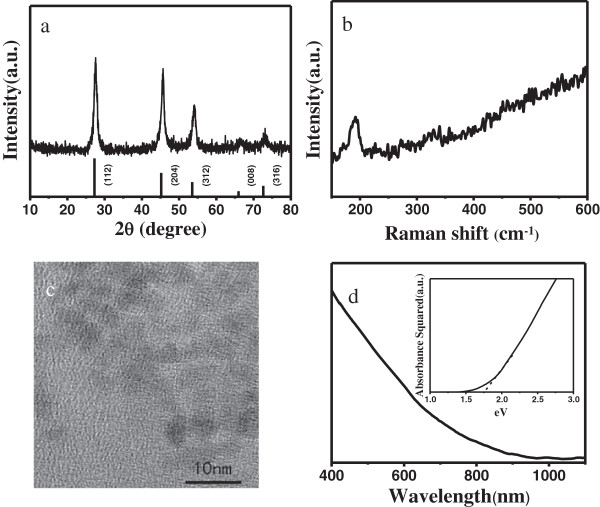
**XRD pattern, Raman spectrum, HRTEM image, and optical absorption spectrum of CZTSe NCs. (a)** XRD pattern of CZTSe NCs. [The standard diffraction lines of tetragonal-phase CTZSe (JCPDS 52-0868) are shown at the bottom for comparison.] **(b)** Raman spectrum of CZTSe NCs. **(c)** HRTEM image of CZTSe NCs. **(d)** Optical absorption spectrum of CZTSe NCs. (The inset shows the bandgap of CZTSe NCs).

Figure 1a shows the XRD pattern of the CZTSe NCs. The diffraction peaks in the XRD pattern appear at 27.3°, 45.3°, 53.6°, 66.3°, and 72.8°, consistent with the (112), (220/204), (312), (400/008), and (316) planes, respectively, which match those of tetragonal-phase CTZSe (JCPDS 52-0868). The diffraction peaks of stoichiometric Cu_2_SnSe_4_ and ZnSe are very similar to those of CZTSe. To ensure our results, Raman scattering is also performed for a more definitive assignment of the structure [26]. Figure 1b shows the Raman spectrum of the CZTSe NCs. One peak at around 192 cm^−1^ is detected, which matches well with that of bulk CZTSe (192 cm^−1^). However, the peaks are slightly broader and shifted with respect to those of the bulk crystal. Broadening of Raman peaks has been observed previously for NCs of other materials and attributed to phonon confinement within the NCs [27]. Both characterizations suggest that pure-phase CTZSe NCs are synthesized.

Figure 1c shows a high-resolution transmission electron micrograph (HRTEM) of CZTSe NCs. The average size of CZTSe NCs is about 3 nm. CZTSe NCs have better dispersibility. Figure 1d shows the UV-vis absorption spectrum of CZTSe NCs and the corresponding bandgap of CZTSe NCs. The bandgap of CZTSe NCs was estimated to be 1.76 eV by extrapolating the linear region of a plot of the squared absorbance versus the photon energy. This is mainly attributed to the small size and quantum confinement effect of CTZSe NCs [28].

**Figure 2:**
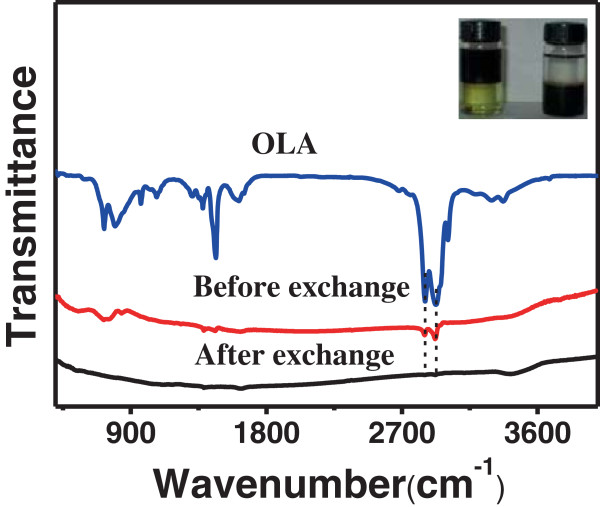
**FTIR spectra of OLA and CZTSe NCs before and after ligand exchange.** The inset shows the colloidal dispersion of CZTSe NCs before and after ligand exchange.

Figure 2 shows the FTIR spectra of OLA and CZTSe NCs before and after ligand exchange. The transfer of CZTSe NCs from toluene to FA resulted in complete disappearance of the peaks at 2,852 and 2,925 cm^−1^ corresponding to C-H stretching in the original organic ligand. As shown in the inset photograph, the two-phase mixture that contained immiscible layers of FA (down) and toluene (up) showed the ligand exchange of CZTSe NCs. After being stirred for about 10 min, complete transfer of CZTSe NCs from a nonpolar solvent (toluene) to a polar solvent (FA) was achieved (Figure 2 inset). The phase transfer can be easily monitored by the color change of toluene (black to colorless) and FA (yellow to black) phases. Black-colored colloidal dispersion of CZTSe NCs capped with organic ligand undergoes the phase transfer from toluene to FA with the inorganic ligand of (NH_4_)_2_S in FA upon exchange of the original organic surface ligand with S^2−^.

**Figure 3:**
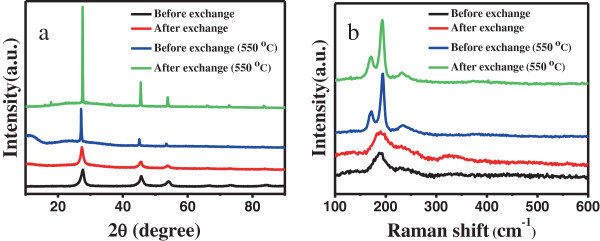
XRD patterns (a) and Raman spectra (b) of CZTSe nanocrystal thin films before and after 550°C selenization.

Figure 3a shows the XRD patterns of CZTSe NC thin films before and after 550°C selenization for 30 min. CZTSe NC thin films were prepared by the dip-coating method. CZTSe NCs were dipped and dried on a silicon substrate from perchlorethylene before ligand exchange and aqueous dispersions after ligand exchange. All the diffraction peaks in the XRD pattern appear at 27.3°, 45.3°, 53.6°, 66.3°, and 72.8°, consistent with the (112), (220/204), (312), (400/008), and (316) planes, respectively, which match those of tetragonal-phase CTZSe (JCPDS 52-0868). These results confirmed that the ligand exchange does not change the structure of CZTSe NCs. The full width at half maximum (FWHM) of the (112) peak before and after ligand exchange is 0.733° and 0.696°, respectively, while the value decreases to 0.222° and 0.120°, respectively, by selenization, indicating a high-quality crystalline structure [29]. From Figure 3a, we can see that the intensity of the diffraction peaks increased largely by selenization after ligand exchange and the FWHM of the (112) peak after ligand exchange was less than that before ligand exchange, indicating the improvement of the crystallinity. XRD patterns show the improvement of the crystallinity after ligand exchange benefits from the removal of the large organic molecules [29].

Herein, Raman spectroscopy was further employed for phase analysis, as shown in Figure 3b. Because (NH_4_)_2_S is used during ligand exchange, the CZTSe nanocrystal thin film shows one weak peak of Cu_2_ZnSnS_4_ at around 333 cm^−1^ after ligand exchange. There are no characteristic peaks of other impurities detected. CZTSe thin films prepared by selenization shows three peaks of CZTSe with Raman shift at 172, 192, and 232 cm^−1^, in agreement with previous reports [30]. These results further confirmed that the ligand exchange did not change the structure of CZTSe NCs. There are no observable secondary phases such as Cu_2_Se, SnSe, and Cu_2_SnSe_3_. The intensity of the Raman peaks increased largely after annealing due to the recrystallization of CZTSe NCs.

**Figure 4:**
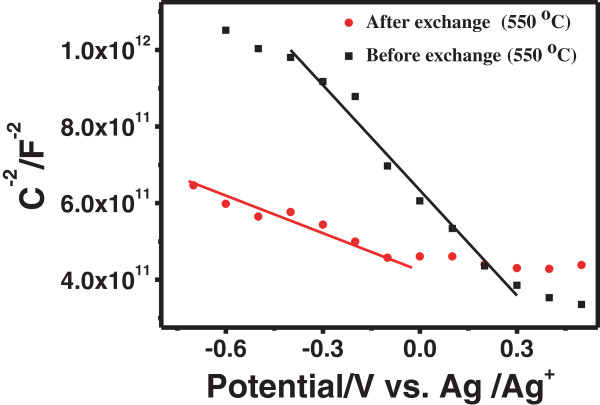
Mott-Schottky plots for CZTSe NC thin films before and after ligand exchange by 550°C selenization.

The resistivity (*ρ*) of CZTSe NC thin films by selenization is listed in Table 1. The resistivity of CZTSe NC thin films before and after ligand exchange is 3.09 and 0.17 Ω cm, respectively. The removal of organic ligand after ligand exchange induces lower resistivity and improves the electronic properties of CZTSe NC thin films. Figure 4 shows the Mott-Schottky plots for the CZTSe NC thin films by selenization before and after ligand exchange in 1 M NaOH solution. The CZTSe thin films show p-type conductivity from the negative slope of the Mott-Schottky plot [31,32]. According to the Mott-Schottky equation [31],

**Table 1:** Energy level and resistivity of CZTSe NC thin films before and after ligand exchange by 550°C selenization

**Samples**	***ρ *** **(Ω cm)**	***E***_**LUMO **_**(eV)**	***E***_**HOMO **_**(eV)**	***E***_**gap **_**(eV)**^**a**^
Before exchange (550°C)	3.09	−3.95	−5.57	1.62
After exchange (550°C)	0.17	−4.37	−5.91	1.54

^a^Determined by CV, |*E*'_ox_ − *E*'_red_|.

(1) $$\frac{1}{C_{\mathrm{sc}}^{2}}=\frac{2}{\epsilon \epsilon_{0}eN_{D}}\left(E-E_{\mathrm{fb}}-\frac{\mathrm{kT}}{e}\right)$$

where *ϵ* is the relative permittivity (dielectric constant) of the CZTSe films, *ϵ*_0_ is the vacuum permittivity, *e* is the elementary charge of an electron, *N*_D_ is the donor density in CZTSe films, *E*_fb_ is the flat-band potential, *k* is the Boltzmann constant, and *T* is the temperature; the carrier concentration is inversely proportional to the slope of 1/*C*^−2^ vs. *E*. It can be seen that the slope of CZTSe films after ligand exchange is smaller than that before ligand exchange, indicating that the carrier concentration increases after ligand exchange and the conductivity of CZTSe NC thin films would be improved.

**Figure 5:**
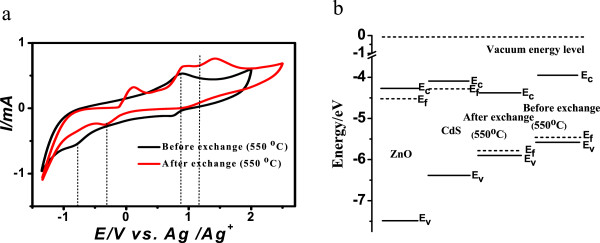
**CV curves of the CZTSe NC thin films and the energy level diagram. (a)** CV curves of the CZTSe NC thin films before and after ligand exchange by 550°C selenization. **(b)** The energy level diagram before the formation of heterojunction in CZTSe solar cells.

The values of HOMO and LUMO energy levels of the materials are crucial for their applications in optoelectronic devices such as solar cells. CV has been utilized to estimate the HOMO energy level (or ionization potential *I*_p_) and the LUMO energy level (or electron affinity *E*_a_) of semiconductor materials [33-36]. The HOMO and LUMO energy levels can be calculated from the onset oxidation potential (*E*'_ox_) and onset reduction potential (*E*'_red_), respectively, according to Equations 2 and 3 [37,38]:

(2) $$E_{\mathrm{HOMO}}=-I_{p}=-\left(E{'}_{\mathrm{ox}}+4.71\right)\;\mathrm{eV}$$

(3) $$E_{\mathrm{LUMO}}=-E_{a}=-\left(E{'}_{\mathrm{red}}+4.71\right)\;\mathrm{eV}$$

where the onset potential values are relative to a Ag/Ag^+^ reference electrode. Figure 5a compares the cyclic voltammograms of NC thin films before and after ligand exchange by selenization. Cyclic voltammograms were carried out in 0.1 M TBAPF_6_/DMF at 50 mV s^−1^ scan rate. As shown in Figure 5a, relative to the Ag/Ag^+^ reference electrode, the onset oxidation and reduction potentials of thin films are 0.86 and −0.76 V, respectively, for the thin film by selenization before ligand exchange and 1.2 and −0.34 V, respectively, for the thin film by selenization after ligand exchange. The bandgap (*E*_gap_) values calculated from the CV measurements are shown in Table 1. The bandgap is about 1.62 eV before ligand exchange. The bandgap is about 1.54 eV after ligand exchange. The removal of large organic molecules is of great benefit to crystallization after annealing treatment [29]. It can be seen in Figure 3a that the film has better crystallinity after ligand exchange by 550°C selenization. Since the bandgap of the poor-crystallized film was much larger than that of the well-crystallized film [39], the bandgap of the film after ligand exchange becomes smaller than that before ligand exchange.

Figure 5b shows the individual energy level of ZnO, CdS, and the absorption layer used for CZTSe solar cells. The HOMO-LUMO levels of the absorption layer by selenization before and after ligand exchange listed in Table 1 are determined from the onset oxidation and reduction potentials according to Equations 2 and 3. It can be seen that the HOMO and LUMO energy levels of the CZTSe layer shift downwards after ligand exchange. If CZTSe solar cells are structured, CZTSe, CdS, and ZnO are in close contact with each other to form a heterojunction. The carrier will transfer between these semiconductors until the three kinds of materials form the unified Fermi level and the heterojunction is in thermal equilibrium state. After ligand exchange, the conduction band of the CdS layer is above that of the CZTSe layer, which is in accordance with the real condition of the CZTSe solar cell. A type I band alignment is more conveniently formed at the CdS/CZTSe interface. This structure acts as the barrier against injection electrons from ZnO to the CZTSe layer, and recombination between majority carriers is not formed [40]. Meanwhile, this structure acts as the barrier against photogenerated electrons in CZTSe, too. Photogenerated electrons cannot cross over the barrier if the height of this barrier at the CdS/CZTSe interface becomes over 0.4 eV. The height should be modestly controlled to keep *J*_sc_ constant [40]. However, before ligand exchange, the conduction band of the CdS layer is below that of the CZTSe layer and a type II band alignment is formed at the CdS/CZTSe interface. This structure will cause recombination between majority carriers at the interface, and the entire recombination increases with increasing absolute value of conduction band difference between CdS and CZTSe layer [40]. As a result, the open circuit voltage of the CZTSe solar cell will become higher after ligand exchange due to the type I band alignment structure and the depression of recombination.

## Conclusions

In conclusion, we synthesized pure tetragonal-phase structure CZTSe NCs with the size of about 3 nm by a facile one-step synthesis. For potential application in CZTSe solar cells, the physical mechanism of utilizing energy level alignment for reducing recombination was discussed in depth after ligand exchange. It was found that the removal of large organic molecules on CZTSe NCs after ligand exchange by S^2−^ decreased the resistivity. The bandgap of the films after ligand exchange by 550°C selenization was also decreased due to better crystallinity. HOMO and LUMO energy levels of CZTSe films both shifted down after ligand exchange, and a type I band alignment structure was more conveniently formed at the CdS/absorption layer interface in CZTSe solar cells. This structure acts as the barrier against injection electrons from ZnO to the CZTSe layer, and recombination will subsequently be depressed. Overall, the cell efficiencies relatively depend on the energy level alignment and ligand exchange will make great contribution in this aspect.

## Competing interests

The authors declare that they have no competing interests.

## Authors’ contributions

XW and DXK participated in the design and coordination of the study. DXK and SXW conceived the study and drafted the manuscript. WHZ and XC participated in the sequence alignment and performed the synthesis and characterization of the obtained CZTSe nanoparticles and films. ZJZ performed the CV measurements. All authors read and approved the final manuscript.
